# A Monocortical Screw for Preventing Trochanteric Escape in Extended Trochanteric Osteotomy: A Simple Solution to a Complicated Problem?

**DOI:** 10.3390/jcm12082947

**Published:** 2023-04-18

**Authors:** Petros Ismailidis, Annegret Mündermann, Karl Stoffel

**Affiliations:** 1 Department of Orthopaedics and Traumatology, University Hospital of Basel, Spitalstrasse 21, 4031 Basel, Switzerland; 2 Department of Clinical Research, University of Basel, 4031 Basel, Switzerland; 3 Department of Biomedical Engineering, University of Basel, 4123 Allschwil, Switzerland

**Keywords:** extended trochanteric osteotomy, ETO, trochanteric escape, revision total hip arthroplasty

## Abstract

Extended trochanteric osteotomy (ETO) is an established method in revision total hip arthroplasty. Proximal migration of the greater trochanter fragment and the resulting non-union of the osteotomy remains a major problem, and several techniques have been developed to prevent its occurrence. This paper describes a novel modification of the original surgical technique in which a single monocortical screw is placed distally to one of the cerclages used for the fixation of the ETO. The contact between the screw and the cerclage counteracts the forces applied on the greater trochanter fragment and prevents trochanteric escape under the cerclage. The technique is simple and minimally invasive, does not require special skills or additional resources, or add to surgical trauma or operating time, and therefore represents a simple solution to a complicated problem.

## 1. Introduction

Total hip arthroplasty (THA) is a successful and established treatment for hip osteoarthritis [1]. The number of people affected by hip osteoarthritis, and thus the number of THAs, has steadily increased over the past decades, and this trend is expected to continue in the future [2]. Because of the increasing number of THAs and the increasing number of elderly people, the number of THA revisions is expected to increase [3]. The removal of well-fixed cemented and non-cemented femoral stems is one of the major surgical challenges in THA revision to treat late THA infection and malpositioned stems that lead to recurrent THA dislocation, as well as to improve acetabular exposure [4]. In these cases, extended trochanteric osteotomy (ETO) is an efficient and popular technique to remove well-fixed stems [5].

ETO has several advantages, including wide exposure, safe cement removal, and low risk of iatrogenic injuries such as canal perforation or intraoperative fractures [6,7,8]. ETO has mid-term survival rates of approximately 90% and is currently considered the technique of choice for revision THA [8]. However, a major problem with ETO is the relatively high incidence of trochanter migration and consequent non-union of the osteotomy [9,10,11]. Non-union rates initially ranged from 1 to 29% in various studies 11 with the most recent evidence reporting a mean non-union rate of 7% [12]. The resulting biomechanical changes have a negative impact on clinical outcomes [9]. Since the initial description of ETO, various surgical methods and new implants have been described with the aim of improving osteotomy fixation and avoiding trochanter migration [13,14,15,16]. None of these methods have been established as a reliable fixation method for preventing trochanteric escape.

This paper presents a novel surgical technique aimed at preventing trochanter migration after ETO. First, the original ETO technique and the biomechanical causes leading to a trochanteric escape are presented and explained using a representative case. Then, the technical details of the new surgical technique and the biomechanical principles that prevent trochanteric escape are presented using a clinical case. Finally, the advantages and disadvantages of this technique are discussed in comparison to other techniques that address the same problem.

## 2. Surgical Technique

### 2.1. Extended Trochanteric Osteotomy

#### 2.1.1. The Surgical Technique

**Figure 1:**
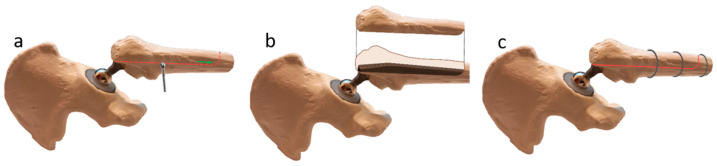
The ETO as described by Younger et al. [17]. (**a**). The posterior and distal part of the osteotomy border is outlined with several drill holes. The osteotomy is performed by connecting the holes with the saw or pencil bur. A cerclage is placed distal to the osteotomy to prevent a femoral fracture. The anterior part of the osteotomy is performed separately with the oscillating saw. (**b**). The osteotomy fragment is mobilized anteriorly with the osteotome. The stem is exposed. (**c**). After changing the stem, the osteotomized trochanter fragment is reduced and fixed with 2 to 3, 2.0 mm cerclage wires or cables.

The ETO technique was originally described by Younger et al. [17]. A posterolateral approach in the lateral decubitus position is used to access the proximal femur. However, a direct lateral approach or a modification of approaches that provide access to the proximal femur is also suitable for ETO. The approach extends distally as far as necessary to access the stem to be removed. The fascia lata and the fascia of the gluteus maximus muscle are split along the incision. The tendinous insertions of the short external rotators are dissected. The posterior capsule is incised, and a flap of the capsule is mobilized to allow access to the joint. The vastus lateralis muscle is mobilized anteriorly subperiostally, and the proximal femur is exposed. A cerclage is placed distal to the osteotomy tip to prevent femoral fracture. The posterior and the distal part of the osteotomy margin are outlined with multiple drill holes (Figure 1a). The most distal point of the osteotomy extends distally to the tip of the prosthesis. The drill points are then connected with the oscillating saw or a pencil bur. The anterior cortex of the femur is perforated with multiple drill holes, entering through the posterior part of the osteotomy and trying to encompass one third of the femoral circumference. The anterior portion of the osteotomy is then performed with the oscillating saw. The osteotomized fragment is mobilized with the osteotome as a single unit (Figure 1b). In our clinic, the osteotomy is performed before dislocating the hip. However, osteotomy after dislocation and either before removal of the femoral component or after removal of the femoral component is also possible. After removal of the stem and placement of the new stem, the osteotomized trochanter fragment is reduced and fixed with 2 to 3 2.0 mm cerclage wires or cables (Figure 1c).

#### 2.1.2. The Postoperative Regime

The patient is allowed to mobilize from day 1 with partial weight-bearing of the operated leg. Flexion is limited to 90°. Active abduction is not allowed as to prevent dislocation of the trochanteric fragment. The use of an abduction orthosis was suggested in the original ETO description [17] but this is not used in our clinic.

### 2.2. The Problem: Trochanteric Escape after ETO

Trochanteric escape is a common complication after ETO [10,18,19]. It is the proximal migration of the osteotomized fragment of the greater trochanter that “escapes” under the fixation with cerclage wires or cables.

#### 2.2.1. Biomechanical Principles of Trochanteric Escape

**Figure 2:**
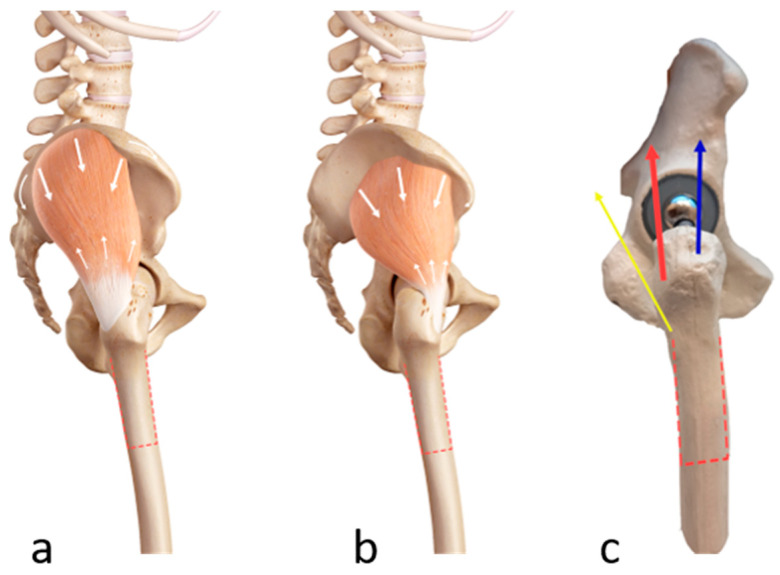
(**a**). Anatomy of the gluteus medius muscle: the gluteus medius muscle originates on the lateral surface of the ilium and attaches to the posterior and lateral part of the superior aspect of the greater trochanter. (**b**). Anatomy of the gluteus minimus muscle: the gluteus minimus originates on the lateral surface of the ilium and attaches at the anterolateral aspect of the greater trochanter. (**c**). Illustration of the direction of the forces applied by the gluteus medius (red) and minimus (blue) muscles on the osteotomized fragment. Contraction of the gluteus medius muscle produces an abduction force to the osteotomized trochanter fragment, whereas contraction of the gluteus minimus muscle produces an abduction and a lesser internal rotation force to the osteotomized trochanter fragment. Together, activation of these two muscles can cause cranial dislocation of the trochanter fragment. The external rotators also attach to the osteotomized fragment and produce an external rotation force (yellow); however, this force is lower than the force of the other two muscles and is not relevant for the trochanteric escape.

The greater trochanter is the attachment site of the gluteus medius and minimus muscles. The gluteus medius muscle originates from the lateral surface of the ilium. It has an anterior posterior and middle portion, all of which attach to the posterior and lateral part of the superior aspect of the greater trochanter. The gluteus minimus muscle originates on the lateral surface of the ilium, lies immediately beneath and just anterior to the gluteus medius, and attaches at the anterolateral aspect of the greater trochanter [20,21,22]. The osteotomized fragment is attached to both muscles. Contraction of the gluteus medius muscle produces an abduction force to the osteotomized trochanter fragment, whereas contraction of the gluteus minimus muscle produces an abduction and lesser internal rotation force to the osteotomized trochanter fragment [21]. Together, activation of these two muscles can cause cranial dislocation of the trochanter fragment if the fixation is not adequate (Figure 2).

#### 2.2.2. Clinical Case

**Figure 3:**
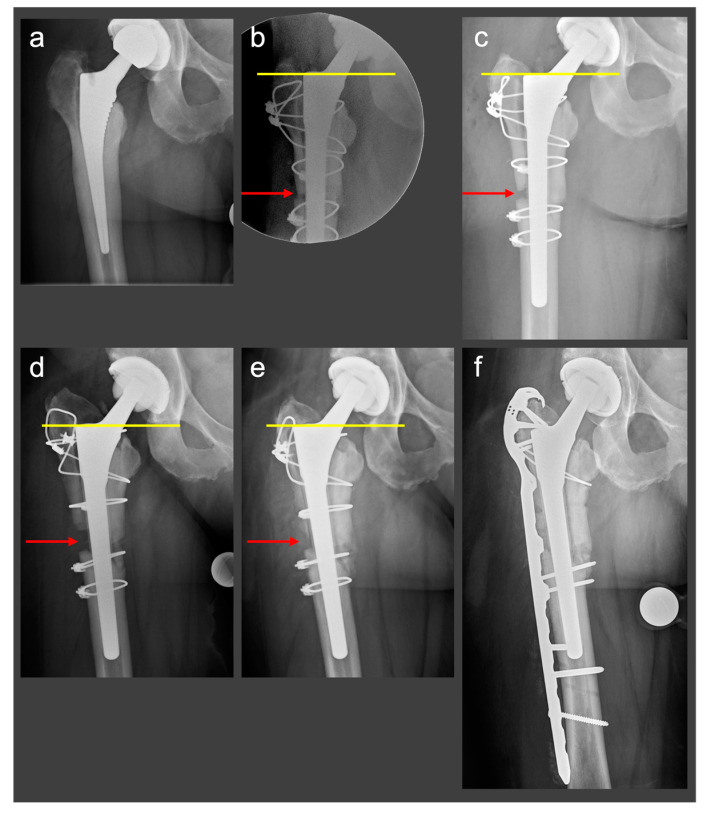
Case of a patient with a trochanteric escape after ETO. (**a**). Two years after implantation of a fully coated non-cemented stem, the patient presented with an early aseptic stem loosening. Metaphyseal radiolucency and the diaphyseal cortical hypertrophy are seen, indicating a lack of metaphyseal osteointegration with a diaphyseal mechanical bone stress. (**b**). Intraoperative images: An ETO was performed for stem removal. The osteotomy was reduced and fixed with three horizontal cable cerclages and one combined vertical and horizontal cable fixation. An intraoperative fracture of the medial cortex occurred. (**c**). Three days postoperatively, after patient mobilization: note trochanteric escape with a loss of reduction of the osteotomy fragment (red arrow) and proximal migration of the trochanter fragment (yellow line outlines the shoulder of the prosthesis, which is now level with the tip of the cerclage). (**d**). Three months postoperatively: note further cranial dislocation of the trochanter fragment causing a larger osteotomy gap (red arrow). The tip of the vertical cerclage is now clearly above the shoulder of the prosthesis (yellow line). (**e**). Five months postoperatively: no healing tendency at the osteotomy gap is seen (red arrow). (**f**). The symptomatic non-union was treated with an open reduction and internal fixation of the fragment with a periprosthetic plate.

A 48-year-old female patient (height 1.7 m, body mass 82 kg, body mass index 28.4 kg/m^2^, no relevant comorbidities) underwent THA for hip osteoarthritis with a fully coated non-cemented stem (twinSys^®^, Mathys AG^®^, Bettlach, Switzerland). After 2 years, a THA revision with stem change had to be performed because of early aseptic stem loosening (Figure 3a). The patient was referred to our institution. An ETO was performed for stem removal, and a distally tapered stem was implanted (Wagner SL stem, Zimmer Inc., Warsaw, Indiana, USA). A cable cerclage was placed distal to the osteotomy to prevent further femur fracture. The osteotomy was reduced, fixed with three horizontal cable cerclages and one combined vertical and horizontal cable fixation [13] (Figure 3b). Intraoperatively, a fracture of the medial femoral cortex occurred. Postoperatively, the patient was allowed to mobilize with partial weight-bearing of the operated leg, flexion was limited to 90°, and active abduction was not allowed. Serial radiographs were obtained intraoperatively and at 3 days and 3 months postoperatively, documenting cranial dislocation of the greater trochanter fragment (Figure 3c,d). Because of the presence of symptomatic non-union, 6 months after the ETO the patient underwent revision surgery as well as open reduction and internal fixation of the fragment with a periprosthetic hook plate (LCP™ periprosthetic proximal femur plating system, Depuy-Synthes, Warsaw, IN, USA).

### 2.3. Novel Surgical Technique: A Monocortical Screw for Preventing Trochanteric Escape

#### 2.3.1. The Surgical Technique

**Figure 4:**
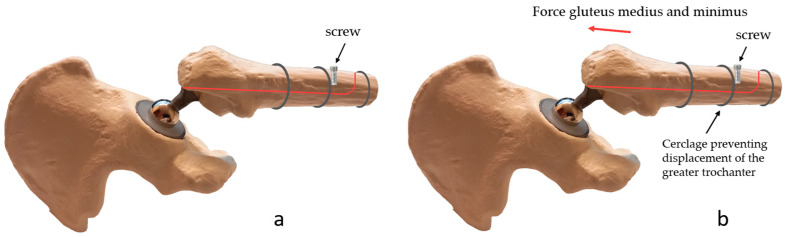
(**a**). Novel surgical technique: a monocortical periprosthetic screw is placed on the trochanter fragment immediately distal to the distal cable cerclage fixing the osteotomy. (**b**). Biomechanical principles that prevent trochanteric escape: The force exerted by the gluteus medius and minimus muscles pulls the trochanter fragment cranially. The screw placed on the trochanter fragment is pulled cranially with the trochanter fragment. The contact of the screw with the distal cerclage prevents the screw and thus the trochanter fragment from moving cranially.

The ETO is performed as described above. The ETO is reduced and fixed with cable cerclages. To prevent cranial dislocation of the trochanter fragment, a monocortical periprosthetic screw is placed under the cable cerclage on the trochanter fragment just distal to the distal cable cerclage fixing the osteotomy (Figure 4a).

#### 2.3.2. Biomechanical Principles

The force applied by the gluteus medius and minimus muscles pulls the trochanter fragment cranially. The screw placed on the trochanter fragment is pulled cranially with the trochanter fragment. However, the contact of the screw with the distal cerclage prevents the screw, and thus the trochanter fragment, from moving cranially (Figure 4b). Dislocation of the fragment is only possible if the cerclage moves cranially along with the screw or if the screw “escapes” below the cerclage. Cranial movement of the cerclage is highly unlikely because of the inverted conical shape of the proximal femur. An “escape” of the screw under the cerclage would only be possible in the case of a loose cerclage.

#### 2.3.3. Clinical Case

**Figure 5:**
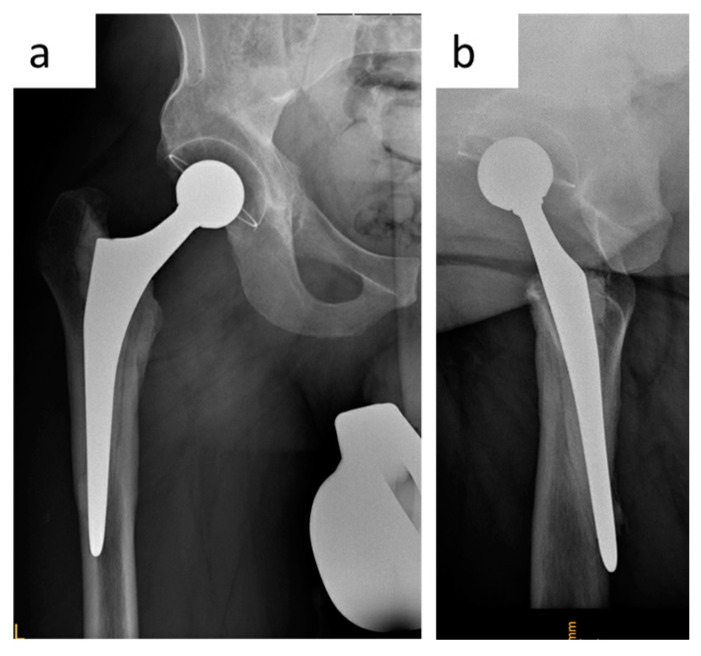
Anteroposterior (**a**) and axial (**b**) views of the right hip before THA revision. The patient had originally received a THA with a polished tapered stem. An intraoperative femoral fracture (diaphyseal cortical perforation, via falsa) went unnoticed and was diagnosed only postoperatively.

**Figure 6:**
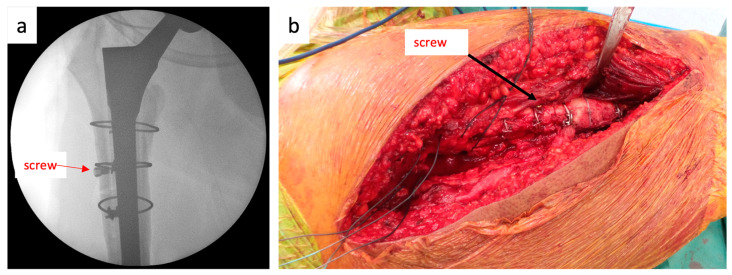
(**a**). Intraoperative fluoroscopic image. Note the screw placed just distal to the cerclage fixing the osteotomy. (**b**). Posterior intraoperative view showing the position of the screw just distal to the cerclage.

**Figure 7:**
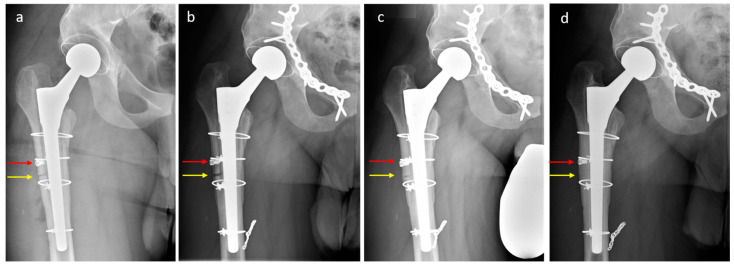
Radiographs 3 days (**a**), 3 months (**b**), 6 months (**c**), and 18 months (**d**) postoperatively documenting the progressive bony union of the osteotomy without dislocation (in the interim, the patient received osteosynthesis of the anterior acetabular column for symptomatic non-union after a previous periacetabular osteotomy and coiling of a pseudoaneurysm of a branch of the profunda femoris artery). Note the position of the screw (red arrow) and the gradual healing of the osteotomy site (yellow arrow).

A 34-year-old patient (height 1.75 m, body mass 95 kg, body mass index 31 kg/m^2^, no relevant comorbidities) underwent THA with a polished tapered (force-closed) stem because of hip osteoarthritis after hip dysplasia previously treated with periacetabular osteotomy. An intraoperative femoral fracture (diaphyseal cortical perforation, via falsa) went unnoticed and was diagnosed only postoperatively (Figure 5). The patient was referred to our institution 6 months postoperatively. He experienced pain in the thigh and groin and was on crutches. A revision THA was performed with a change of the stem through an ETO. A distally tapered stem was implanted (Wagner SL stem, Zimmer Inc., Warsaw, Indiana, USA). Two cable cerclages were placed distal to the osteotomy to prevent femur fracture. The osteotomy was reduced and fixed with two cable cerclages with a 5.0 mm monocortical periprosthetic screw (LCP™ periprosthetic proximal femur plating system, Depuy-Synthes, Warsaw, Indiana, USA) placed distal to the distal cerclage (Figure 6). Postoperatively, the patient was allowed to mobilize with partial weight-bearing of the operated leg, flexion was limited to 90°, and active abduction was not allowed. Serial radiographs 3 days and 3, 6, 12, and 18 months postoperatively document the bony union of the ETO without evidence of fragment dislocation (Figure 7). At the 18-month follow-up, the patient was satisfied with the outcome of surgery, was able to walk without crutches, and began to play sport. He still had residual pain but reported a clear improvement compared to before the stem change.

## 3. Discussion

Trochanteric escape and the resulting non-union remain a major problem after ETO, occurring in up to 29% of cases [10]. Several authors have explored conservative and surgical options to avoid this complication.

### 3.1. Trochanteric Escape: Available Solutions

#### 3.1.1. Partial Weight-Bearing, Abduction Restriction

The original publication describing ETO [17] already recognized the risk of trochanteric escape and suggested a postoperative rehabilitation scheme trying to prevent the trochanteric escape. The authors suggested partial weight-bearing and restriction of abduction with an abduction brace. Abduction braces have been shown to be effective in limiting the range of motion, although not to the extent promised by the brace settings [23]. Although no study to date has specifically addressed their ability to prevent trochanter fragment dislocation after ETO, abduction braces have been shown to be ineffective in preventing THA dislocation [23,24,25,26], and they are not suitable for obese patients [26]. From a biomechanical perspective, restricting abduction alone does not reliably protect the trochanter fragment from the forces exerted by the gluteal muscles because the maximal contractile force exerted by the abductor muscles occurs during the single leg stance phase [27,28], when the gluteus medius restricts the lowering of the pelvis. Therefore, partial or non-weight-bearing until the bony union is reached is essential to reduce the forces acting on the trochanter fragment. However, several studies have shown that patients do not reliably bear partial weight even when instructed to do so by a physiotherapist [29]. Therefore, limiting weight-bearing and abduction alone seems inadequate to protect the osteotomy.

#### 3.1.2. Surgical Options to Prevent Trochanteric Escape

Several authors have attempted to modify the original ETO method to prevent trochanteric escape. Mei et al. [30] performed a systematic review of the various fixation options. In addition to wires and cables, the three main categories of available trochanteric fixation methods are cable plate systems, claw or locking plates, and trochanteric bolts.

Horizontal wire and cable cerclages were described in the original ETO publication [17], and modifications of the wire or cable positioning have been developed since then [30]. Horizontal cerclages—while easy to perform—can allow trochanteric escape below the cerclages. To address this problem, modifications in cerclage placement, such as combined vertical and horizontal cable fixation or passing the cerclage through the trochanter fragment, have been described [13]. Nevertheless, breakage or loosening of the cerclage is a common complication that can lead to loss of fixation [30]. Moreover, cases have been reported in which fractured portions of the cerclages have migrated to sites distant from the osteotomy site, such as between the THA components [31], in the popliteal fossa [32], or even intravascular [33].

Cable plate systems and claw or locking plates have been shown in vitro to be more biomechanically stable than wires and cerclages [19]. However, they are known to be associated with pain and bursitis trochanterica, and have higher reoperation rates compared with wires [30]. They also require specialized hardware and are more technically demanding and expensive.

Trochanteric bolts are specialized devices that have been reported only in calcar replacement prostheses [30], and therefore are not suitable for regular use in ETO cases. Furthermore, mechanical complications such as fracture of the trochanter by the bolt and breakage or disassembly of the bolt have been reported. Overall, they have higher rates of bursitis and reoperation compared with wires and should be reserved for special cases requiring calcar replacement [30].

### 3.2. A Monocortical Screw for Preventing Trochanteric Escape: Advantages and Disadvantages

The method described here only requires the placement of a single monocortical screw distal to a cerclage. It is easy to perform, not time-consuming, does not require special orthopaedic devices, and cannot cause hardware-related complications. Therefore, this technique is an easily applicable modification of the original ETO technique making reasonable use of resources. The major disadvantage of this technique is that it is completely dependent on the integrity of the cerclage and will fail in the event of cerclage loosening, slippage of the cerclage over the screw, or breakage of the cerclage. A conventional screw with a washer may be an alternative to the periprosthetic screw used in this case. The washer would make the screw head more prominent, reducing the possibility of the cerclage slipping over the screw.

### 3.3. The Importance of Bony Union in ETO

Two studies found no difference in revision rates between ETOs with trochanteric non-union and ETOs in which bony union has been achieved [10,11]. Nevertheless, the function of the hip abductor mechanism is only possible with a trochanteric union. Not surprisingly, a non-union of the ETO has a negative effect on clinical outcome [9].

The abductor muscle group consists of the primary and secondary abductor muscles. Primary abductors are the gluteus medius, gluteus minimus, and tensor fasciae latae muscles. The secondary hip abductors are the piriformis, sartorius, and rectus femoris muscles. Dislocation of the trochanter fragment means for a loss of function of the two primary abductors, the gluteus medius and minimus muscles. Complete loss of abductor function results in Trendelenburg gait [18]. Additionally, even minor deficits in abductor strength negatively affect lower limb function, causing knee osteoarthritis [34,35], patellofemoral pain syndrome [36], or even chronic lower back pain [37]. Overall, adequate abductor muscle function has a positive effect on physical function and prevents limping after THA [35,38], whereas impaired abductor muscle function increases the risk of falls [39]. In summary, healing of the bony trochanter fragment and this preservation of abductor mechanism function is crucial in revision THA.

## 4. Conclusions

A single monocortical screw placed distal to one of the cerclages used for fixation of the ETO is a technical modification of the original ETO with the aim of preventing dislocation of the trochanter fragment. The contact between the screw and the cerclage counteracts the forces acting on the greater trochanter fragment and prevents trochanteric escape under the cerclage. The technique is simple and minimally invasive, requires no special skills or additional resources, nor adds to surgical trauma or operating time, and therefore may provide a simple solution to a complicated problem. In this article, we described this novel technique, and the biomechanical principles that govern it through, based on a case report. Future publications, including a series of patients treated with this technique, are needed to evaluate its clinical potential.

## Data Availability

Not applicable.
